# A Review of Capabilities and Scope for Hybrid Integration Offered by Silicon-Nitride-Based Photonic Integrated Circuits

**DOI:** 10.3390/s22114227

**Published:** 2022-06-01

**Authors:** Frederic Gardes, Afrooz Shooa, Greta De Paoli, Ilias Skandalos, Stefan Ilie, Teerapat Rutirawut, Wanvisa Talataisong, Joaquín Faneca, Valerio Vitali, Yaonan Hou, Thalía Domínguez Bucio, Ioannis Zeimpekis, Cosimo Lacava, Periklis Petropoulos

**Affiliations:** 1 Optoelectronics Research Centre, University of Southampton, Southampton SO17 1BJ, UK; a.shooa@soton.ac.uk (A.S.); gdp1n18@soton.ac.uk (G.D.P.); i.skandalos@soton.ac.uk (I.S.); sti1g14@soton.ac.uk (S.I.); teerapat.rutirawut@soton.ac.uk (T.R.); wanvisa.talataisong@soton.ac.uk (W.T.); v.vitali@soton.ac.uk (V.V.); yaonan.hou@soton.ac.uk (Y.H.); t.dominguez_bucio@soton.ac.uk (T.D.B.); izk@soton.ac.uk (I.Z.); pp@orc.soton.ac.uk (P.P.); 2 Instituto de Microelectrónica de Barcelona, IMB-CNM (CSIC), Campus UAB, 08193 Barcelona, Spain; joaquin.faneca@imb-cnm.csic.es; 3 Electrical Computer and Biomedical Engineering, University of Pavia, 27100 Pavia, Italy; cosimo.lacava@unipv.it

**Keywords:** silicon nitride, modulation, nonlinear photonics, laser integration

## Abstract

In this review we present some of the recent advances in the field of silicon nitride photonic integrated circuits. The review focuses on the material deposition techniques currently available, illustrating the capabilities of each technique. The review then expands on the functionalisation of the platform to achieve nonlinear processing, optical modulation, nonvolatile optical memories and integration with III-V materials to obtain lasing or gain capabilities.

## 1. Introduction

In the last decade, the accelerated development of photonic integrated circuits (PICs) based on silicon-compatible materials has been mainly driven by the need to develop devices that can fulfil the high demands of optical communications and data interconnects in the near-infrared (NIR) [1,2,3]. However, as the integrated platforms have matured, the use of PICs have broadened to applications in which some of the intrinsic properties of silicon pose a challenge. As a result, there has been an increased interest in exploring different CMOS compatible materials that can complement the functionality of silicon, such as amorphous silicon, silicon oxynitride, silicon nitride, aluminium nitride (AlN) and alumina (Al_2_O_3_) [4,5,6,7,8].

Amongst these materials, silicon nitride (SiN) is a mature CMOS compatible platform that has gained significant attention for the demonstration of linear, nonlinear and active integrated photonics devices [9] due to its flexible optical properties that can be tailored for a wide range of integration schemes and applications. SiN is suitable for broadband applications since it has a wide optical bandgap (2.7–5 eV) that sets the lower limit of its transparency window to the ultraviolet (UV) regime extending it all the way to the mid-infrared ( 5 μm). The material also offers a compromise between level of integration and flexible dimension control as it has a modest refractive index that can be tuned between 1.7 and 3.1 to provide a good optical confinement in the near-infrared (NIR) with a high tolerance to both surface roughness and dimensional variations that enable one to attain ultralow propagation losses [10,11,12]. Additionally, it has a low thermo-optic coefficient ($10^{-5}$), which makes it attractive for the realisation of devices that are highly sensitive to temperature variations. Finally, it exhibits a third-order Kerr nonlinearity with negligible two-photon absorption (TPA) in the NIR which makes it suitable for nonlinear applications.

This review article presents the recent progress on the integration of the SiN platform in a variety of interesting research areas. This includes the demonstration of waveguides with sub-dB propagation losses in the C-band ( 1550 nm) for all-optical processing and the use of custom SiN films with a high silicon concentration to achieve large Kerr coefficients for nonlinear optics. Additionally, it covers the integration of SiN with a range of materials such as electro-optic polymers, lithium niobate (LN), barium titanate oxide (BTO), graphene, TMD semiconductors and transparent conducting oxides (TCOs) to demonstrate optical modulators. It further discusses the integration of SiN with phase change materials (PCMs) and a postfabrication device trimming technique as strategies to achieve nonvolatile photonic devices. Finally, it incorporates the integration of III-V materials and SiN waveguides as a means to achieve efficient lasers on a silicon substrate. The vast subject of sensing and in particular gas sensing and biological sensing, which is the subject of intense research for chemical functionalisation and sensing through evanescent field interaction, is not covered in this review as it has been covered extensively elsewhere [13,14].

## 2. Low-Loss Silicon Nitride Materials

SiN films are typically deposited using either high-temperature low-pressure chemical vapour deposition (LPCVD) or low-temperature plasma-enhanced chemical vapour deposition (PECVD). Both methods rely on chemical reactions in the gas form that result in hydrogenated amorphous films with a composition that tends to depart from the stoichiometric form of the material (Si_3_N_4_). Hence, these films are characterised by the presence of hydrogen bonds (Si-H and N-H), which act as absorption centres that can lead to undesirable losses in the NIR, particularly in the C-band centred at 1550 nm. The main difference between the two methods is that LPCVD layers are deposited at high temperatures (>800 °C), while PECVD films are deposited at much lower temperatures (<400 °C).

Although PECVD films enable the back-end-of-line integration with materials that are sensitive to temperature, layers deposited through LPCVD are by far preferred for the growth of SiN films for single-layer photonic integrated circuits since high quality layers with a high density and high uniformity can be achieved with a relatively low processing cost and high throughput. Moreover, high-temperature LPCVD processes have the energy required to break the hydrogen bonds that can increase the optical losses in the NIR, enabling the realisation of devices with losses <1 dB/cm.

In fact, LPCVD SiN waveguides with ultralow propagation losses <0.1 dB/m at 1550 nm and a record low loss of 0.045 dB/m at 1580 nm have been demonstrated since 2011 [15,16,17]. To achieve these results, the typical strip waveguide geometry that provides a moderate optical confinement with a thickness ranging between 200 and 400 nm needs to be replaced with a high aspect ratio geometry with a thickness below 100 nm. By using such a geometry, the optical confinement of the waveguide is reduced allowing more than 85% of the mode to travel in the SiO_2_ cladding. Therefore, the large mode area reduces the scattering losses by minimising the mode interaction with the sidewall roughness of the waveguide, allowing a decrease of the propagation losses from values close to 1 dB/cm attained with the moderate confinement geometry to the sub-dB scale [18,19,20].

Although the low-confinement approach drastically reduces the propagation losses of the waveguides, it increases the footprint of the fabricated devices since radii >5 mm are required to reduce the bending losses. As a result, waveguides with a small effective area and high confinement are necessary to enable tighter bends for large scale photonic integrated systems [21,22]. Furthermore, they are critical to achieve high optical intensity, dispersion engineering for nonlinear processes and a reduced birefringence to potentially allow for the fabrication of polarisation-independent devices [18,21,22].

However, as the modes within such thick waveguides are highly localised in the core, both the intrinsic losses of the material and the scattering losses produced at the interfaces of the waveguide have a stronger impact than radiation losses on the minimum propagation losses that can be realised. Moreover, the growth of SiN layers with thickness >400 nm poses a fabrication challenge as they tend to crack due to the tensile stress of the material, especially if they are deposited with a high-temperature LPCVD process [23].

Several approaches have been proposed to demonstrate thick SiN layers with low propagation losses. As previously discussed, the propagation losses are the result of the scattering losses that occur due to surface roughness, the absorption produced chemical impurities and defects within the material, and the radiation losses directly related to the confinement of the light. Most of the proposed methods focus on reducing the absorption losses of the material layer and the scattering losses through advanced fabrication techniques, since the radiation losses of the highly confined geometries tend to be negligible. In this section, we discuss some of the methods that have been used to achieve losses <1 dB/cm and <2 dB/cm using LPCVD and PECVD SiN layers.

### 2.1. Thermal Cycling

**Figure 1:**
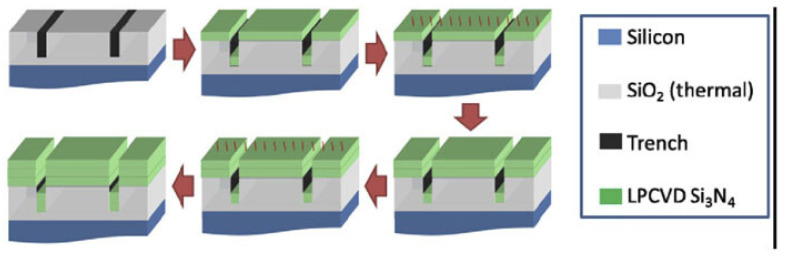
Schematic thermal cycling process. Trenches are formed and then, a 350 nm film of Si_3_N_4_ is deposited via LPCVD and annealed at 1200 °C. The same process is repeated to achieve the desired thickness. Reproduced from [22] under a CC BY 4.0 license.

Since 2013, Lipson et al. have demonstrated a subtractive method based on thermal cycling and stress relieving mechanical trenches to produce stoichiometric Si_3_N_4_ layers with thickness up to 910 nm on 100 mm oxidised silicon wafers (Figure 1). In this approach, the stress of the films is alleviated by mechanically scribing a series of trenches on the top SiO_2_ surface around the edges of the wafer [24]. These trenches prevent the formation of cracks that can propagate across the wafer, creating crack-free regions in which photonic devices can be fabricated.

The growth of the LPCVD layer is performed in a multistep process to minimise the internal stress of the layers. Firstly, a 350 nm film is deposited and then annealed at 1200 °C for 3 h in an Ar atmosphere to reduce its hydrogen concentration in order to minimise its absorption losses. Afterwards, both steps are repeated to reach the required thickness [22]. The top roughness of the surface layer can be further reduced by means of a chemical mechanical polishing step (CMP) before patterning the devices using electron beam lithography (EBL) and inductively coupled plasma reactive ion etching (ICP-RIE) using a CHF_3_/O_2_/N_2_ chemistry [22,25]. Finally, to minimise the contribution of the cladding to the propagation loss of the devices, the wafer is cladded with a 250 nm layer of high-temperature oxide (HTO) followed by 2 μm of PECVD oxide. Using this fabrication method, the authors have not only demonstrated thick Si_3_N_4_ films with low-thickness nonuniformity <1%, but they have realised 1800 × 910 nm waveguides with propagation losses as low as 0.04 dB/cm at 1550 nm [22,24].

### 2.2. Photonic Damascene Reflow Process

**Figure 2:**
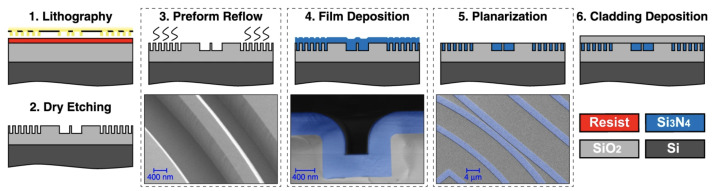
Damascene reflow process steps including lithography, preform dry etching, preform reflow, LPCVD Si_3_N_4_ deposition, planarisation and cladding deposition. Reproduced from [21] under a CC BY 4.0 license.

In 2015, Epping et al. introduced an innovative additive fabrication process that can be used to fabricate waveguides with a thickness up to 900 nm by depositing a thick Si_3_N_4_ layer into trenches patterned on a 100 mm thermally oxidised wafer [26]. After further optimisation, this method evolved into the photonic Damascene reflow process illustrated in Figure 2 [27,28,29,30]. In this technique, waveguide trenches and a dense stress-release pattern are written onto a wafer by means of EBL and photolithography. The full pattern is then etched through reactive ion etching (RIE) using a thin amorphous silicon layer as a sacrificial hard mask which is then stripped. Before depositing the Si_3_N_4_ film, a reflow step of the oxide preform is performed by heating the substrate at 1250 °C to reduce the sidewall roughness, which can introduce additional scattering losses without deforming the waveguide. The LPCVD Si_3_N_4_ film with a thickness up to 1.5 μm is then deposited in order to fill completely the previously etched trenches. The device waveguides are then obtained by removing the excess Si_3_N_4_ by means of CMP to achieve a planar and polished surface. Once the waveguides are obtained, the wafer is annealed at 1150 °C to reduce the absorption losses of the material. Finally, the substrate is cladded with a layer of tetraethyl orthosilicate (TEOS) followed by a low-temperature oxide layer (LTO). This optimised method has allowed the demonstration of 2000 × 600 nm waveguides with propagation losses of 0.05 dB/cm at 1550 nm [29]. Furthermore, this process has been further optimised to provide high-yield and full wafer-scale fabrication of low-loss devices on 100 mm substrates [21]. To achieve this, EBL has been replaced by DUV stepper lithography, which enables the increase of the throughput and the reproducibility of the process. Additionally, an etch-back process based on dry etching and CMP has been introduced to decrease the wafer-scale thickness variation of the Si_3_N_4_ layer (<3%).

### 2.3. Multistep Annealing

**Figure 3:**
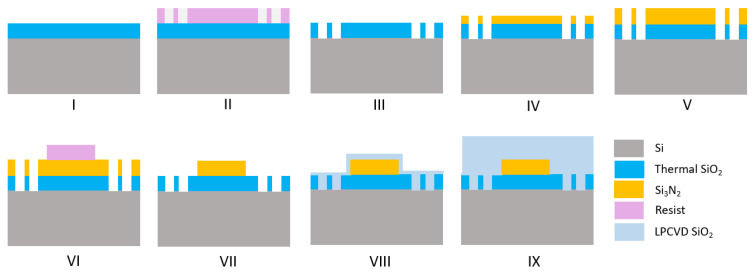
Multistep annealing process flow: (**I**) thermal oxidation, (**II**) photolithography, (**III**) SiO_2_ HF etch, (**IV**) Si_3_N_4_ deposition and annealing at 1100 °C, (**V**) second Si_3_N_4_, (**VI**) E-beam lithography, (**VII**) Si_3_N_4_ etch, (**VIII**) LPCVD SiO_2_ deposition and annealing at 1100 °C and (**IX**) PECVD SiO_2_ deposition. Reprinted with permission from [31], ©2019 The Optical Society.

In 2018, Ye et al. proposed another subtractive fabrication method illustrated in Figure 3, that relies on multiple annealing steps to achieve films with thicknesses >600 nm on 76 nm silicon wafers with a 3 μm thermal oxide [31]. In this method, the stress-release trenches and the thermal cycling process described previously are adopted to reduce the crack formation. After transferring the stress-release pattern with direct laser writing, the Si_3_N_4_ layer is deposited in an LPCVD furnace in two steps, in between which the deposited film is annealed at 1100 °C for 3 h in a N_2_ ambient to minimise its hydrogen content. Later on, the devices are patterned on the layer by means of EBL and ICP-RIE with a CHF_3_/O_2_ chemistry. After etching, the wafer is annealed again at 1100 °C for 3 h in N_2_ to further reduce the hydrogen. The waveguides are then covered with a 500 nm layer of low-pressure TEOS deposited at 710 °C which is then annealed at 1100 °C in N_2_ to increase its density. Finally, the devices are cladded with 2 μm of PECVD oxide. Following this method, the authors have realised 1800 × 645 nm waveguides with propagation losses of 0.03 dB/cm at 1550 nm [31]. Furthermore, amongst the methods described previously, this technique has been the only one that has demonstrated Si-rich SiN waveguides of a cross-section of 1850 × 600 nm, exhibiting propagation losses of 0.4 dB/cm at 1550 nm [32].

### 2.4. Twist-and-Grow

**Figure 4:**
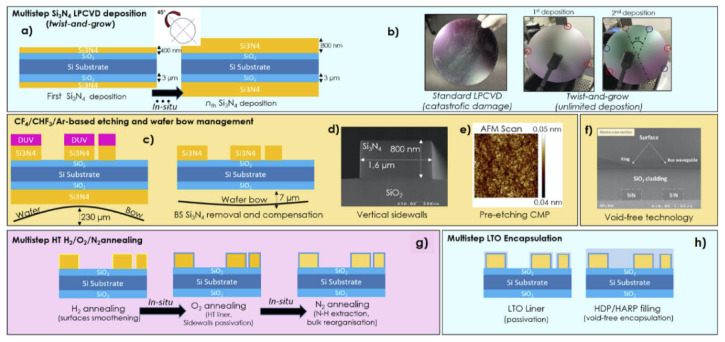
Schematics of the twist-and-grow and multiple-step chemical-physical annealing process: (**a**) twist-and-grow deposition of Si_3_N_4_, (**b**) images of the grown Si_3_N_4_ with and without twist-and-grow, (**c**) fluorocarbon dry etching and wafer bow management, (**d**) example of a Si_3_N_4_ waveguide with sidewall angle <2 deg after CMP, (**e**) AFM micrograph before CMP, (**f**) SEM cross-section of the encapsulated waveguides, (**g**) multistep H_2_/O_2_/N_2_ in situ annealing and (**h**) multistep encapsulation. Reprinted with permission from [33], ©2019 The Optical Society.

In 2018, El Dirani et al. developed a deposition technique known as twist-and-grow illustrated in Figure 4, that has been used to produce thick Si_3_N_4_ layers (>1 μm) in a standard large-format fabrication line [33]. The fabrication begins with a blanket 200 mm silicon substrate with a 3 μm thermal oxide layer, on which a 800 nm thick Si_3_N_4_ film is deposited in two steps using the twist-and-grow technique [34,35]. In this method, the layers are deposited by means of LPCVD at 780 °C with a deposition rate of <2 nm/min that enables the production of films with minimal hydrogen content. In between steps, the wafer is cooled down to 630 °C to eliminate stress-related cracks and rotated by 45° to redistribute the uniaxial stress that can significantly bow the wafer. The wafer then goes through a CMP step to reduce the surface roughness before patterning the waveguides using DUV lithography and inductively coupled plasma etching (ICP) with a tailored CF_4_/CHF_3_/Ar that minimises sidewall roughness. The resist and any etching residues are removed with an O_2_ plasma and the surface is cleaned with a H_2_SO_4_ solution before selectively etching the Si_3_N_4_ on the backside of the wafer to alleviate the tensile stress that can appear after patterning to ensure that the wafer can continue its fabrication in the process line. In order to reduce the propagation losses, the patterned wafer is annealed at 1100 °C for a few minutes in H_2_ to smoothen the sidewalls, at 1200 °C in O_2_ to encapsulate the waveguides with a native oxidation to reduce scattering centres, and at 1200 °C for 3 h in N_2_ to drive out the hydrogen impurities that contribute to the absorption of the material in the NIR. As a last step, the waveguides are encapsulated with a TEOS and a LTO layer deposited by high-density plasma-enhanced chemical vapour deposition (HDP-ECVD). Using this fabrication process the authors have not only demonstrated 1600 × 800 nm waveguides with propagation losses of 0.035 dB/cm at 1560 nm [33], but they have also demonstrated a largely scalable process with high reproducibility that has potential for applications that require thicker layers.

### 2.5. Summary

Table 1 summarises the main features of the processes that have been used to demonstrate SiN layers with losses in the sub-dB/cm regime. All these methods have achieved losses <0.1 dB/cm by applying a combination of stress-release patterns, multistep deposition processes, multiple annealing steps and CMP planarisation. However, although they provide very low losses, none of these techniques are compatible with the CMOS back-end-of-line which is necessary to achieve the multilayer integration required for more complex photonic integrated circuits, as they all have steps with high-processing temperatures (>400 °C). Moreover, all the methods have mostly focused on optimising the Si_3_N_4_ stoichiometric composition (n = 2.0), except for the multistep annealing process that has also demonstrated low-loss Si-rich SiN films (n = 2.07). This narrow range of refractive indices poses limitations for nonlinear applications that require higher refractive indices to achieve stronger nonlinear effects [11,36] and for the demonstration of devices operating at shorter telecom wavelengths which often benefit from lower refractive indices to minimise propagation losses and phase errors [37,38].

## 3. Si-Rich Silicon Nitride for Nonlinear Photonics

Variation of the gas composition of the film-forming reactants during the silicon nitride (SiN) deposition process affects not only the linear part of the refractive index, but also the nonlinear part. This provides an additional degree of freedom in the design of all-optical signal processing devices based on third-order (Kerr) nonlinear effects [39,40,41]. Si-rich SiN layers of a varying refractive index can be grown by increasing the amount of SiH${}_{4}$ during the material deposition process. As reported in the literature [39,42], an increase in the silicon concentration allows a greater nonlinear Kerr coefficient to be realised, however, at the expense of typically higher absorption losses. Therefore, a proper material composition can be selected depending on the specific requirements of the target application. For example, low-loss ( 0.4 dB/cm ) Si-rich SiN with a refractive index of 2.07 at 1550 nm (slightly greater than that of stoichiometric Si_3_N_4_) was used to fabricate microresonators with high quality factors (Q ∼$0.8\times 10^{6}$) in the C and L bands, allowing the generation of broadband coherent frequency combs in this platform [32]. On the other hand, despite its higher propagation losses (3–10 dB/cm), ultra-silicon-rich nitride (USRN, refractive index equal to 3.1 at 1550 nm) emerged as a promising platform for the development of highly compact nonlinear devices operating at low optical power levels, thanks to its nonlinear refractive index being two orders of magnitude greater than that of stoichiometric Si_3_N_4_ [36,43,44,45,46,47]. Table 2 summarises the main features of several Si-rich SiN platforms, with different refractive indices, demonstrated in the literature. In the following subsections, we report the most recent developments in Si-rich SiN-based nonlinear optics, focusing our attention on some specific examples showcasing the flexibility of this material platform for the design of nonlinear devices, manifested by the tunability of its linear and nonlinear optical properties.

### 3.1. Tunable Nonlinear Optical Properties

We review here a comprehensive experimental study to show the dependence of the nonlinear optical properties on the Si-rich SiN material composition [39]. Specifically, the nonlinear performance of three layers deposited on a thermal SiO${}_{2}$ substrate with different refractive index was investigated, whose properties are summarised in Table 3. Fully etched waveguides with different widths (in the range 0.5–1.5 μm) were written onto the different layers and characterised in the measurement campaign. Light coupling with optical fibres was achieved by means of grating couplers (GCs) and tapered edge-couplers (ECs).

**Figure 5:**
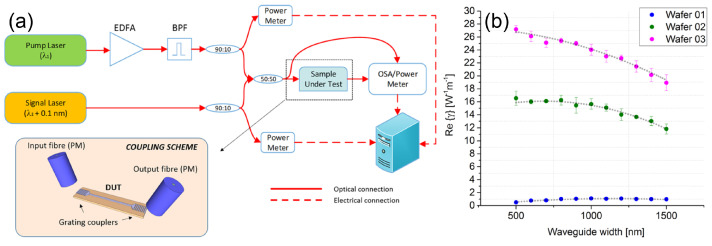
(**a**) CW-FWM experimental setup. BPF: bandpass filter; EDFA: Erbium-doped fibre amplifier; OSA: optical spectrum analyser. (**b**) Experimentally measured Reγ for each waveguide geometry, on each Si-rich SiN layer. Reproduced from [39] under a CC BY 4.0 license.

The third order nonlinear response of an optical waveguide can be generally expressed in terms of the nonlinear parameter γ:

(1) $$\gamma \left(\omega \right)=\omega \frac{n_{2}}{cA_{eff}}+i\frac{\beta_{TPA}}{2A_{eff}}$$

where ω is the angular frequency, $n_{2}$ is the nonlinear refractive index, *c* is the speed of light, $A_{eff}$ is the effective area and $\beta_{TPA}$ is the two-photon absorption (TPA) coefficient. The real part of Equation (1) (also known as Reγ) describes the Kerr response of the optical waveguide and introduces a nonlinear phase shift to the propagating light. The imaginary part of the Equation (also known as Imγ) represents the TPA response, that accounts for the power-dependent nonlinear losses that the optical signal undergoes during propagation. The values of Reγ for the different Si-rich SiN layers were experimentally measured by using a continuous-wave (CW) four-wave-mixing (FWM)-based scheme [39], whose experimental setup is shown in Figure 5a. A pump laser (wavelength $\lambda_{1}$ = 1550.11 nm, optical power ranging from 20 mW to 2 W at the waveguide input GC) was amplified by a polarisation-maintaining Erbium-doped fibre amplifier (PM-EDFA) and then was sent to an optical band pass filter (BPF) to filter out the EDFA-generated amplified spontaneous emission (ASE) noise. A 50:50 fibre coupler was employed to combine the pump with a weaker CW optical signal (the signal optical power was always kept at least 10 dB below the pump power level) generated by a tunable external cavity laser (ECL). GCs were used to couple the two optical waves into the waveguides under test. A FWM-induced idler at a new frequency was generated in the waveguide and the three signals were coupled back to a PM fibre by means of an output GC. By recording optical spectra for different pump power levels, the Reγ coefficients of the fabricated waveguides were derived using the following equation:

(2) $$Re\gamma =\frac{\sqrt{P_{i}\left(L\right)/P_{s}\left(L\right)}}{\eta P_{p}\left(0\right)L_{eff}}$$

where $P_{i}\left(L\right)$ and $P_{s}\left(L\right)$ are the idler and signal optical powers measured at the waveguide output, respectively, $P_{p}\left(0\right)$ is the pump power at the waveguide input, $L_{eff}$ is the nonlinear effective length and η accounts for the phase-mismatch resulting from chromatic dispersion [49]. In the considered case, since the signal and pump waves were placed relatively close to each other in wavelength (Δλ <0.15 nm), the effect of dispersion can be neglected and it is therefore possible to consider η = 1.

The results of the nonlinear measurement campaign are reported in Figure 5b. It can be seen that layers 02 and 03 show around an order of magnitude greater nonlinear Kerr response than layer 01. The reason for this enhancement can be found in the increased material nonlinearities resulting from the presence of additional Silicon in the material composition. Specifically, by increasing the silicon concentration, the linear refractive index of the material increases and this is accompanied by an increase in the nonlinear Kerr coefficient as well, in line with the empirical rule developed by Miller [50].

The imaginary part of the nonlinear coefficient, Imγ, was then measured by means of pulse-transmission experiments [39,51]. A fibre mode-locked laser centred at 1550 nm (pulse duration: 0.5 ps; repetition rate: 20 MHz) was employed as the light source. Unlike the previously described CW experiments, the light was coupled in and out of the integrated waveguides by using tapered ECs, in order not to impose any spectral distortion on the laser pulses. The TPA coefficients $\beta_{TPA}$ of the various Si-rich SiN layers were estimated by measuring the average power at the waveguide output as a function of the input peak power. Under the assumption of a hyperbolic-secant pulse temporal profile (which is the nominal pulse shape of the optical source used in these experiments), the average output power $P{\left(L\right)}_{avg}$ can be expressed as a function of the input peak power $P{\left(0\right)}_{peak}$ through the following equations [51]:

(3) $$P{\left(L\right)}_{avg}=\frac{ln(\sqrt{\sigma }+\sqrt{\sigma +1})}{\sqrt{\sigma (\sigma +1)}}P{\left(0\right)}_{avg}e^{-\alpha L}$$

(4) $$\sigma =\frac{\beta_{TPA}}{A_{eff}}L_{eff}P{\left(0\right)}_{peak}$$

where $P{\left(0\right)}_{avg}$ is the average input power, α is the propagation loss coefficient and *L* is the waveguide physical length. TPA coefficients $\beta_{TPA}$ (and hence Imγ) for the three Si-rich SiN layers were derived using Equations (3) and (4) and the measured values for three different waveguide widths (500, 700 and 1000 nm) are reported in Table 4.

The experimental results showed that both layers 01 and 02 were not significantly affected by TPA-related losses, even when relatively high optical power levels (>10 W peak power) were employed. Conversely, when the silicon content in the material was further increased (as in the case of layer 03), TPA-related effects started to appear, suggesting that this Si-rich SiN composition was not suitable for high-power low-loss applications.

### 3.2. FWM-Based Frequency Conversion in Multimode Si-Rich Silicon Nitride Waveguides

The discussion in the previous section has shown that Si-rich SiN layers can be properly engineered to obtain a high Kerr nonlinear response with no TPA-related losses. This allows waveguides to be operated at Watt-power levels, unlike silicon itself, which is strongly limited by TPA-related effects.

**Figure 6:**
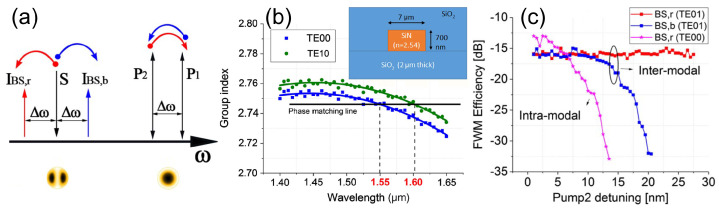
(**a**) Operation principle of the dual-pump IM-BS-FWM process: energy is transferred directly from the signal (S) to two idlers ($I_{BS,r}$ and $I_{BS,b}$) and between the two pumps ($P_{1}$ and $P_{2}$). (**b**) Numerically simulated group index curves for a Si-rich SiN waveguide (n = 2.54 at 1550 nm), with the waveguide cross-section shown in the inset. (**c**) BS-FWM efficiency measured as a function of pump $P_{2}$ detuning for the intermodal and intramodal configurations. Reproduced from [40] under a CC BY 4.0 license.

In this configuration, the two pumps were placed into the TE00 mode of the waveguide, while the signal and the generated idlers were in the TE10 mode. As in any FWM-based process, efficient nonlinear conversion takes place only when the phase-matching condition among the different waves is satisfied. In particular, efficient IM-BS-FMW can be achieved over a wide bandwidth whenever the inverse group velocity curve of one mode is a frequency-shifted replica of the other mode [52]. The propagation properties of the two modes were engineered by acting on two parameters: the waveguide cross-section and the refractive index of the core Si-rich SiN material. As shown in Figure 6b, the phase-matching condition between the TE00 and TE10 modes can be satisfied over a large bandwidth by considering $\lambda_{TE00}$ = 1550 nm and $\lambda_{TE10}$ = 1601 nm. Moreover, this waveguide design can be used to efficiently suppress one idler in order to have a unidirectional FWM process. In fact, one limitation of the BS-FWM mechanism is its bidirectionality, which can result in the generation of unwanted idlers at new frequencies [53,54]. Numerical simulations confirmed that by setting $P_{1}$ at a fixed wavelength equal to 1550 nm and changing the pump-to-pump separation Δω by varying the position of $P_{2}$, it is possible to achieve a constant conversion efficiency for $I_{BS,r}$ for a large $P_{2}$ detuning range (>50 nm). Conversely, the generation of $I_{BS,b}$ is hindered in this scenario, and it takes place only over a narrow Δω range [40]. A measurement campaign was also carried out, whose results are shown in Figure 6c. The wavelengths of the two pumps were initially set at $\lambda_{P1}$ = 1550 nm and $\lambda_{P2}$ = 1551 nm, while the signal wavelength was set at $\lambda_{S}$ = 1601.5 nm. The IM-BS-FWM conversion efficiency was measured for $I_{BS,r}$ and $I_{BS,b}$ by gradually detuning $\lambda_{P2}$ towards longer wavelengths. As predicted by numerical simulations, the conversion efficiency of $I_{BS,r}$ remained constant at a value of around −15 dB across a large $\lambda_{P2}$ detuning range, while $I_{BS,b}$ was no longer detectable for a $P_{2}$ detuning value equal to 20 nm.

### 3.3. Nonlinear Optics in Ultra-Silicon-Rich Nitride Platform

Ultra-Silicon-rich Nitride (USRN) is typically grown at a low temperature ( 250 °C) using inductively coupled chemical vapour deposition and is characterised by a high silicon content compared to nitrogen (Si${}_{7}$N${}_{3}$), with a refractive index equal to 3.1 at 1550 nm [36,43]. The nonlinear optical properties of this material were characterised by open and closed aperture z-scan measurements [55]. A nonlinear refractive index $n_{2}$ equal to $2.8\times 10^{-17}$m${}^{2}$/W was measured at 1550 nm, which is 100× larger than the $n_{2}$ value of stoichiometric Si_3_N_4_ [56,57] and more than 10× larger than the $n_{2}$ value of all the other Si-rich SiN platforms reported in the literature (see Table 2 for comparison). This allows the realisation of highly efficient and compact nonlinear devices, with a total waveguide length shorter than 1 cm, operating at low optical power levels. However, the high silicon concentration makes it particularly challenging to achieve low propagation losses compared to the case of Si-rich SiN compositions with lower refractive indices. Open aperture z-scan measurements showed that TPA is absent in the C-band, with three-photon absorption being the dominant contributor to nonlinear losses. The high linear and nonlinear refractive index of USRN allows one to achieve high values for the waveguide nonlinear parameter (Reγ equal to 500 W${}^{-1}$m${}^{-1}$ for a waveguide cross-section of 0.30 × 0.55 nm^2^ [43]), making it possible to obtain large nonlinear conversion/amplification at relatively low pump power levels [36]. For example, an optical parametric gain of 42.5 dB was demonstrated using a pulsed pump and a CW signal by degenerate-FWM in a high-optical-confinement USRN waveguide ( 4.5 dB/cm propagation losses) operating in the anomalous dispersion regime in the 1550 nm wavelength region [43]. Cascaded FWM with gain down to the third generated idler was also observed, with peak parametric gains measured for the first, second and third idlers equal to 36.2, 21.2 and 7.7 dB, respectively. A further enhancement of the material nonlinearity was achieved by exploiting the slow-light effect in USRN photonic crystal waveguides, with the demonstration of an optical parametric gain per unit length of 333 dB/cm [58]. In this study, FWM experiments were carried out with a pulsed pump and a CW signal in a 97 μm long USRN photonic crystal waveguide (22 ± 2 dB/cm propagation losses in the slow light region) with a measured optical parametric signal gain and idler conversion efficiency of 3 dB and −1 dB, respectively. The nonlinear efficiency of the USRN material was also proven in the observation of temporal soliton dynamics, specifically in the soliton-effect temporal compression and fission of optical pulses [48,59]. High-order solitons are generated in optical media characterised by the simultaneous presence of anomalous dispersion and nonlinearity, and they evolve periodically during propagation, experiencing a temporal narrowing at the beginning of each soliton period. This property can be used to realise strong temporal compression of optical pulses, which allows the generation of ultrashort pulses and the increase of the pulses’ peak powers. Then, the high-order soliton compression process was exploited to achieve a 8.7× temporal compression of 2 ps optical pulses using a low pulse energy equal to ∼16 pJ in a 7 mm long USRN waveguide (characterised by 3 dB/cm propagation losses) [48]. To the best of our knowledge, this is the largest soliton-effect temporal compression demonstrated on an integrated photonic waveguide to date. Wideband nonlinear spectral broadening of input fs laser pulses based on self-phase modulation was also demonstrated in USRN add-drop ring resonators (broadening factor of two) and waveguides (broadening factor of around three per 1 mm length) [46]. More recently, thermo-optically tunable nonlinear spectral broadening was reported using an USRN device consisting of a 3 mm long cladding-modulated Bragg grating and a 7 mm long channel waveguide. An increase in the bandwidth of the output pulse spectrum from 69 to 106 nm was measured by decreasing the temperature from 70 °C to 25 °C [60]. Wide supercontinuum generation exceeding 0.6 of an octave at 1550 nm was also demonstrated in a 7 mm long USRN waveguide using 500 fs optical pulses [61].

While several demonstrations of FWM-based nonlinear applications were reported in USRN waveguides using pulsed pumps, the propagation losses of this platform are still too high to allow efficient CW-FWM experiments in centimetre-long waveguides. Opportunities to further improve this material platform and reduce the propagation losses exist, such as the adoption of established protocols for low-loss Si_3_N_4_ waveguide fabrications such as high temperature annealing or the use of the photonic Damascene reflow process. In order to reduce Si-H and N-H bonds-related losses, recipes that make use of alternative gases other than silane could be investigated. For example, the use of dichlorosilane gas (SiH${}_{2}$Cl${}_{2}$) already showed promising results towards a further optimisation of this material platform [62,63]. The reduction of the propagation losses in combination with the large Kerr coefficient of the USRN platform could allow the demonstration of efficient CW-operated phase-sensitive parametric amplifiers and frequency combs with sub-μW threshold power.

## 4. High-Speed Modulators on the Silicon Nitride Platform

Optical modulators are essential devices used to imprint an electrical signal on an optical signal by means of an optical carrier. Modulation mechanisms can be categorised as: electro-optic (EO) [64], magneto-optic (MO) [65], thermo-optic (TO) [66] and acousto-optic (AO) [67].

**Figure 7:**
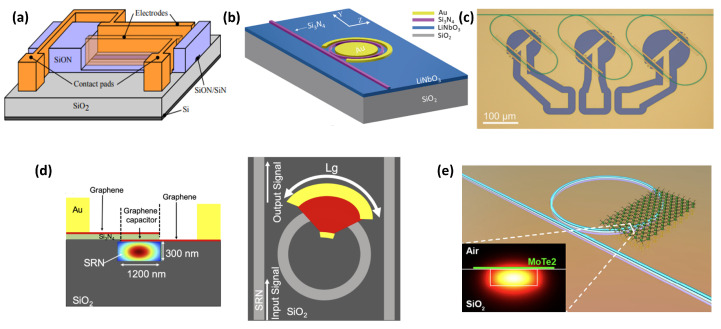
(**a**) Three-dimensional schematic of EO-polymer integrated in SiN-SiON platform; reproduced from [82] under a CC BY 4.0 license. (**b**) Schematic of the tunable hybrid SiN-LN microring resonator; reprinted with permission from [83], ©2019 The Optical Society. (**c**) Top view of hybrid BTO-SiN racetrack resonator; reproduced with permission from [84], ©2019 American Chemical Society. (**d**) Schematic cross-section of the hybrid waveguides based on graphene capacitors and top view of a microring resonator partially covered by a graphene capacitor; reproduced from [85] under a CC BY 4.0 license. (**e**) Schematic of Si${}_{3}$N${}_{4}$ ring resonator covered with monolayer-MoTe${}_{2}$; reprinted with permission from [86], ©2021 American Chemical Society.

On-chip high-speed optical modulators have been developed since 2004 by Lipson et al. [68] and Liu et al. [69], achieving critical milestones towards unlocking the potential of silicon for optoelectronic applications. This was further refined to achieve modulation speeds of up to 50 Gb/s by employing carrier-depletion-based pn-junction structures [70,71,72]. Nonetheless, with the emergence of SiN as a complementing platform to the functionality of silicon photonics, the need for high-speed, low-power optical phase modulation has shifted from one platform to the other. Considering the dielectric properties of SiN, optical modulation through charge carriers is not possible [73]. Thus, the heterogeneous integration of materials with native EO properties on SiN is a necessary route to obtain modulation. These materials should preferably be compatible with back-end-of-line (BEOL) integration to enable multilayer processing of a number of different materials. Such materials can be electro-optic polymers (EO-polymer) [74,75], lithium niobate (LiNbO${}_{3}$) [76,77], barium titanate oxide (BTO) [78,79], electro-optic lead zirconate tantanate (PZT) [80], 2D materials such as graphene and transition metal dichalcogenides (TMDs) and transparent conducting oxides (TCOs) [81] (Figure 7). The review of the heterogeneous integration of materials enabling the formation of high-speed optical modulators is presented in the following sections.

### 4.1. Electro-Optic Polymer-Based Phase Change Modulators

Optical modulators based on the integration of EO-polymers with SiN waveguides provide flexibility and simplicity for BEOL fabrication [10,87]. Therefore, a number of research works on EO-polymer modulators based on SiN have been proposed and demonstrated over the past decade. Nevertheless, the lower index contrast of SiN compared with Si resulted in a challenge linked to obtaining an optimum trade-off between modulation efficiency and low absorption loss, due to the interaction of the optical mode with the electrodes. In the initial state of EO-polymers, chromophores are naturally random align. Thus, its Pockels coefficient is zero as deposited and requires an activation process described as polling for the modulation purpose. The polling of EO-polymer can be achieved by applying a strong electric field through the polymer in the region surrounding the modulating waveguide whilst the polymer is heated with the temperature close to the glass transition temperature. To simplify the process, a polling process is generally performed by using the device modulation electrodes [88,89]. An in-plane electrode, where the electrodes are located on the waveguide plane, is therefore preferred in order to greatly reduce the complexity of fabrication [90]. The overlap of the mode confined in the waveguide with the polymer and the strength of the electric field all play an important role in the performance of the device, which can be extracted by calculating an overlap integral factor of the optical power in the polymer or overlapping factor of the optical field and modulation electric fields within the polymer [91,92,93]. Thus, the research and study on the design of electrode are of interest for the EO-polymer modulation.

The use of EO-polymers in optical modulators has been developed for more than a decade. The characteristic of modulators based on EO-polymers including the waveguide design, frequency of modulation and figure of merit have been summarised in Table 5. It can be seen from the literature that EO-polymer modulators based on SiN waveguides (900 V·cm) have a higher Vπ · L compared with the Si based waveguide (0.008 V·cm) approach. To improve the performance of EO-polymer modulators based on SiN waveguides, the design of SiN slot waveguides and the space-charge effect of the silicon substrate have been studied and simulated in 2021 [94]. The result in this work shows that by controlling the polling process and the mode confinement, a minimum Vπ · L of 1.47 V·cm can be achieved.

### 4.2. Lithium Niobate Modulators

Traditional electro-optic off-the-shelf optical phase modulators used in long-haul telecom applications are based on in-phase/quadrature (IQ) electro-optic modulators that encode information in the phase and amplitude domain [97]. The fundamental building block of such IQ modulators are the optical phase modulators based on lithium niobate (LN) as they feature a relatively strong Pockels effect EO coefficient tensor of 30.8 pm/V [98], broadband operation with a wide transparency window (0.4–5 μm), excellent chemical and mechanical stability and a low thermo-optic coefficient of $10^{-5}$ [99]. Classic modulation waveguiding structures are based on titanium diffusion of LN wafers and annealed proton exchange, which create a low index contrast (0.02), resulting in a weak optical confinement and high bending radius. This prevents high photonic circuit integration, resulting in devices with a length in excess of 3 cm to achieve 2π phase modulation [100,101]. With the emergence of new fabrication techniques such as single-crystal LN films formed by crystal ion slice [102] and the development of platforms such as LN on insulator (LNOI) offering a high index contrast in the range of 0.7, bending radii below 20 μm are now possible [103].

Hybrid integration of the LNOI platform with SiN is an attractive process as opposed to the monolithic LNOI platforms, in which the waveguiding structures are defined by the direct etching of LN, resulting in slanted waveguides and no CMOS compatibility [104]. Recently, remarkable progress has been achieved in the SiN/LNOI hybrid platform. Ahmed et al. demonstrated a high-efficiency modulator with a 3 dB bandwidth of 30 GHz and an ER of 27 dB using a strip-loaded waveguide configuration [83]. Similarly, a racetrack resonator configuration has shown improved performance over its ring resonator (RR) counterpart due to the change in crystal direction along the optical waveguide path, achieving 2.8 pm/V tunability and an intrinsic Q-factor of $1.3\times 10^{5}$ [105]. Nonetheless, the refractive index of stoichiometric Si_3_N_4_ is lower than that of LN, which results in a high optical confinement in the LN layer and a high bending radius. To solve this, Huang et al. [106] demonstrated the integration of Si-rich SiN-films into LNOI, showing a wide range of devices including a Mach–Zehnder modulator in push–pull configuration capable of high-speed modulation of up to 120 GBaud without digital compensation and a 3 dB bandwidth of 100 GHz.

Despite the promising recent results, the nature of LNOI wafers falls behind foundry-level silicon nitride photonic wafers sizes (200–300 mm) because no epitaxial deposition process or monolithic integration is available and, as a result, heterogeneous integration of such EO materials is seen as the mainstream path of functionalising this platform. Most commonly, deployed in Si photonics, the bonding of thin-film LN-fabricated wafers to SiN waveguides has also been demonstrated [27,107] opening a wide range of chip-level nonlinear applications [108]. The lack of a carrier effect in the SiN-platform results in the lack of active photonics devices, thus cointegration of multiple materials is required [109]. One of the promising technologies to realise this is microtransfer printing which allows for the integration of micrometre-sized material sections or devices processed separately on the same die [110]. Vanachere et al. have shown the successful integration of LN with LPCVD SiN-deposited thin layers, owing to a half-wave voltage-length product of 5.5 V·cm and insertion losses of 7 dB [111]. Despite the technical challenges associated with the integration of LN with SiN, it can enable applications such as LiDAR [112] and quantum computing [113].

### 4.3. Barium Titanate Oxide (BTO) Modulators

Improving the performance of optical phase shifters, switches and modulators is of paramount importance for the realisation of neuromorphic computing circuits [114]. The heterogeneous integration of materials such as electro-optic polymers, LN and PZT has previously been explored, albeit each having problems such as temperature degradation [115] and a large footprint as a result of a relatively small EO coefficient. Recently, the re-emergence of BaTiO${}_{3}$ (BTO), grown using molecular beam epitaxy and wafer bonding fabrication processes on silicon substrates, has shown a very large Pockels coefficient in excess of (900 pm/V) [116]. The limiting factor of BTO EO devices is the high optical propagation losses ranging from 40 to 600 dB/cm caused by the absorption of the hydrogen-rich thin layer of STO, which, once it is annealed, has shown propagation losses as low as 6 dB/cm. Nonetheless, silicon nitride ridge waveguide ring resonator structures have been demonstrated by Ortmann et al., showing a modulation efficiency of 0.3 V·cm, an extrapolated power consumption of 100 nW/FSR and the ability to electrically tune the effective refractive index in the order of $10^{-3}$ which is capable of compensating for unavoidable fabrication imperfections [84]. This results in a combination of ultralow-power refractive index tuning via the Pockels effect of the BTO, low optical losses of SiN thin films ( 1 dB/m) and a wider transparency window, encompassing visible wavelengths when compared to similar BTO-Si waveguide platforms. Lastly, the benefit of integrating SiN thin films in BTO is the lack of mobile charge carriers due to the insulating nature of SiN, which can negatively impact the EO performance of the modulator [117]. While there has been a limited number of devices demonstrated on the SiN platform, they hold the potential to enable applications such as neuromorphic systems [118,119,120,121,122], the capabilities of which have so far been mostly limited by the power consumption and footprint needed for active tuning.

### 4.4. Modulators in 2D Materials

The need for a higher bandwidth and lower optical loss has led to significant research efforts beyond silicon photonics, directed at creating compact, cost-effective and fast light modulation devices [123]. In recent years, graphene and 2D materials (transition metal dichalcogenides (TMDs) and black phosphorus) have attracted increasing attention due to their controllable electronic and optoelectronic properties that can enable new conceptual applications for photonic integrated circuits based on Si and SiN material platforms [124]. Two-dimensional materials exhibit functional properties such as a broadband spectral range, high nonlinearity and strong light–matter interaction and have been previously fabricated using methods such as mechanical exfoliation [125], liquid exfoliation [126] and by chemical vapour deposition (CVD) [127].

#### 4.4.1. Graphene

Graphene consists of a flat monolayer of carbon atoms placed in a two-dimensional (2D) honeycomb lattice structure and has been intensely studied since 2004 when it was firstly introduced by Novoselov et al., due to its controllable electronic and optoelectronic properties [128]. The electroabsorption optical transition in graphene can be controlled by tuning by shifting the electronic Fermi level from the Dirac point [129]. Graphene-based waveguide-integrated optical modulators possess several key advantages such as: broadband operation, high speed operation and compatibility with CMOS processing [130].

In 2013, a hybrid structure using a monolayer of graphene on top of a partially etched SiN waveguide was demonstrated showing a mode power attenuation (MPA) as low as 0.066 dB/μm [131], as well as the demonstration of building blocks such as RRs and Mach–Zehnder interferometers (MZIs) showing excellent broadband operation and 40 dB extinction ratios. Later in 2015, an RR-based electro-optic modulator (EOM) using graphene SiN was demonstrated [132], showing a 30 GHz bandwidth with modulation efficiencies of 1.5 dB/V. Furthermore, Abdollahi Shiramin et al. theoretically proposed a double-layer graphene modulator integrated either on top or within a silicon nitride waveguide core and predicted MPAs of 0.12 dB/μm and 0.026 dB/μm, respectively, with a 23 dB extinction ratio and 2.5 GHz modulation bandwidth [133]. Similarly, a four-graphene-layer-based electroabsorption modulator (EAM) based on double-stripe SiN waveguides has also been investigated by Meiyong Fan et al., showing a high modulation bandwidth of 30.6 GHz, with a switching voltage of 3.818 V and a power consumption of 780.5 fJ/bit [134]. The bandwidth of such devices can be extended further by tuning the SiN layer refractive index. Using this approach, Faneca et al. have shown a silicon rich double-layer graphene EAM design based on RRs with bandwidths exceeding 62.41 GHz, an extinction ratio of 16.5 dB and energy consumption of 0.3 pJ/bit [85].

Attractive features of optical phase modulators such as polarisation insensitivity has also been explored [135], with a reported 3 dB bandwidth of 101 GHz, a power consumption of 271 fJ/bit and a modulation length of 20 μm. Following the demonstration of single high-performance devices, graphene can therefore be integrated to SiN to serve a wide range of applications, for cryogenic operations [136], ultrahigh data rate (Tbit/s) transmission [137] and beam steering architectures for LiDAR [138].

#### 4.4.2. TMDs

Monolayer group VI TMDs are composed of a single layer of semiconductors and two layers of chalcogen atoms placed in a trigonal crystal structure [139]. Intrinsically, the limiting factor of graphene is the lack of an energy bandgap which limits its applicability to applications where a semiconductor behaviour is required. The bandgaps of TMD monolayers are found in the visible to near-infrared optical spectrum regions [140], which makes these monolayers promising materials for efficient emission, modulation and detection on-chip, complementing waveguiding platforms with a compatible transparency window such as SiN.

The interfacing of MoS${}_{2}$ on the SiN platform was demonstrated by Guohua Wei et al. in 2014, showing evanescent coupling from the RR optical mode to the monolayer with an absorption of 850 dB/cm [141]. Yang. et al. have reported an all-optical modulator capable of achieving ERs of up to 10 dB in the visible range using a WS${}_{2}$ layer and a pump light of 532 nm, associated with a probe emitting at 640 nm [142]. Optical modulation in the NIR on a SiN RR has also been achieved by Ipshita Datta et al. using a doped WS${}_{2}$ monolayer by gating ionic liquid, resulting in carrier densities of up to $7.2\pm 0.8\times 10^{-13}$ cm^−2^, high modulation efficiencies (V${}_{\pi }L$) of 0.8 V·cm, a 3 dB modulation bandwidth of 0.3 GHz, along with a FOM (Δn/Δk) of 125 [143]. The gating-induced electrorefractive effect has also been extended to MZIs, showing similar modulation performance to RRs as well as the use of other TMDs such as MoS${}_{2}$ in a parallel plate capacitor configuration with a modulation efficiency of 0.88 V·cm. An order of magnitude modulation efficiency improvement has recently been achieved by Zexing Zhao et al., using a wet transfer method on MoS${}_{2}$ layers showing a V${}_{\pi L}$ of 0.09 Vcm and a tuning efficiency of 5.8 pm V${}^{-1}$ [144]. Other TMDs such as molybdenum ditelluride (MoTe${}_{2}$) have also been integrated in damascene-fabricated SiN thin-films [86], with devices such as RRs showing Q-factors of 3 × 10${}^{6}$ over the O and E telecommunication bands.

### 4.5. TCO-Based ENZ Modulators

Modulation technologies based on transparent conducting oxides (TCOs) in the epsilon-near-zero (ENZ) regime are also potentially compatible with the silicon nitride platform (SiN). TCOs constitute a class of materials that includes indium tin oxide (ITO), aluminium- and gallium-doped zinc oxide (AZO and GZO), along with other less common oxides. These materials combine transparency at visible wavelengths and high electrical conductivity [145]. Because of these properties, TCOs have already been applied to LCD and OLED displays [146] and touchscreens [147], and research is being conducted on their use in next-generation photovoltaic devices [148,149,150]. These materials can also be used to create efficient absorption-based optical modulators. By exploiting the tunability of their plasma frequency, their permittivity can be changed from a positive to a negative value, resulting in a transition between semiconductor-like behaviour and metal-like behaviour. TCO-based ENZ modulators are usually implemented by creating a carrier accumulation at the interface between the TCO and a dielectric, when a voltage bias is applied.

The thickness of the accumulation layer, where the effect of the change in permittivity on absorption is highest, is usually limited to less than one nanometre [151,152]. The overlap of the optical mode with this region is therefore one of the main factors that determine the overall performance of the device. For this reason, ENZ modulators usually need to be combined with plasmonic waveguides, which enhance the confinement of the mode and decrease its effective mode area. This type of modulator can theoretically achieve very high speeds (up to terahertz) [153], compact footprint (down to less than one micron) [154] and generally good performance [155,156]. However, most fabricated devices have only been able to operate at much lower speeds, mostly in the kilohertz range [157,158]. Thanks to improvements in design and fabrication processes, speeds of a few gigahertz have been proven to be possible, maintaining a footprint of less than 10 μm [81]. This result makes ENZ modulators a promising solid-state monolithic alternative to other modulators that are compatible with the SiN platform. This compatibility is guaranteed by the fact that modulation only occurs in the TCO layer, and the results are almost independent from the material that forms the waveguides, once the optical mode is converted into a plasmonic mode and coupled into the active region. To the best of our knowledge, TCO-based modulators have not yet been tested on the silicon nitride platform. However, the feasibility of integrating TCO layers and SiN has been proven for different applications [159,160], and simulated modulators show a similar behaviour to those integrated into the standard SOI platform [161,162].

### 4.6. Summary

To conclude, Table 6 provides a summary of the typical optical modulation parameters such as modulation bandwidth, modulation efficiency and optical loss of different modulation technologies associated with SiN photonics. When compared to the other modulators, the 2D materials provide the best modulation efficiency with reported values as low as 0.09 V·cm. Whereas the Si-rich SiN-LNOI MZI show a modulation bandwidth up to 100 GHz with sub-dB/cm optical losses.

## 5. Nonvolatile Photonics and Trimming

Reconfigurable photonic integrated circuits [163,164] have gained tremendous interest in different areas such as neuromorphic computing [114,165], microwave photonics [166,167,168], quantum systems [169,170,171,172] and field-programmable photonic gate arrays (FPPGAs) [173]. The concept of low-power to zero-power reconfigurability requires nonvolatile materials enabling amplitude or phase modulation with low-power consumption. In this section, two different approaches to target nonvolatile photonic building bocks using a silicon nitride waveguide are presented: (i) phase change materials and (ii) postfabrication laser trimming.

### 5.1. Phase Change Materials

Phase change materials (PCMs) are one of the main candidates for nonvolatile reconfigurable photonic integrated circuits as they show a high refractive index or high absorption contrast in their optical properties. Taking advantage from the rewritable optical media and resistive memories, the mature PCMs technology is seen as a route for complementing the CMOS-compatible silicon nitride photonic process [174] to achieve nonvolatile integrated systems. PCMs allow the possibility of switching between two principal states, an amorphous and a crystalline phase, but intermediate states of crystallisation are also feasible. PCMs demonstrate long-term stability at room temperature [175,176,177] with cyclability in the order of $10^{15}$ [178,179,180]. The PCM switching times oscillate depending on the material and the switching mechanism selected (optical or electrical); the fastest reports are in the order of nanoseconds or less [181,182].

**Figure 8:**
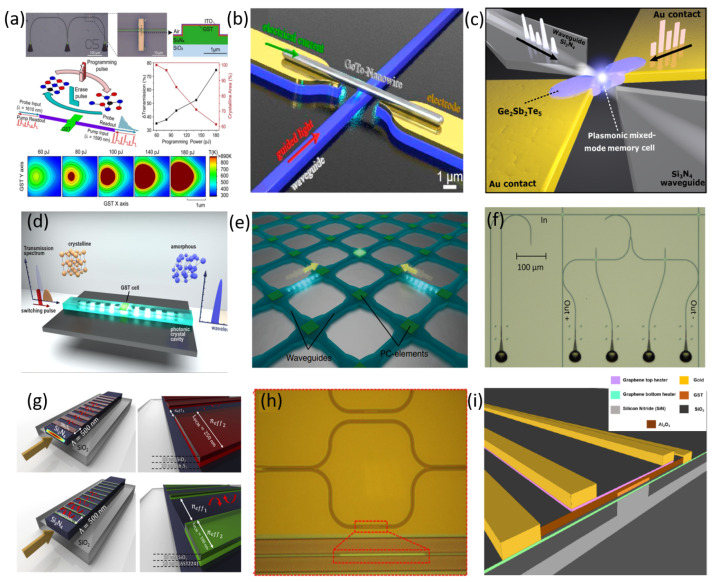
(**a**) Illustration of an all-optical nonvolatile photonic integrated memory based on GST; reproduced from [190] under a CC BY 4.0 license. (**b**) GeTe-based nanowire on a SiN waveguide; reprinted with permission from [192], ©2016 American Chemical Society. (**c**) A combination of GST with partially etched SiN rib waveguide-integrated plasmonic nanogap; reproduced from [184] under a CC BY 4.0 license. (**d**) GST-based SiN photonic crystal nanobeam cavity; reprinted with permission from [193], ©2018 American Chemical Society. (**e**) Image of an all-optical calculator, a photonic abacus; reproduced from [195] under a CC BY 4.0 license. (**f**) SiN waveguides with GST acting as on-chip matrix multiplication as part of a computationally specific integrated photonic hardware accelerator (tensor core); reproduced from [197] under a CC BY 4.0 license. (**g**) Schematic of low-loss nonvolatile SiN photonic-integrated Bragg grating based on Sb_2_S_3_ and GSST; reproduced from [204] under a CC BY 4.0 license. (**h**) Microscope image of a SiN MZI with a Sb_2_S_3_ cell deposited in one of the arms; reproduced from [207] under a CC BY 4.0 license. (**i**) Schematic of an integrated photonic graphene microheater for phase change chalcogenides on SiN building blocks; reproduced from [215] under a CC BY 4.0 license.

The pseudo-binary phase change material Ge_2_Sb_2_Te_5_ (GST) is one of the main nonvolatile materials, and is commonly employed in various silicon-based applications, such as optical switches [183,184], memories [185,186,187], wavelength division multiplexers [188] and optical neural networks [189]. In 2015, for the first time, Ríos et al. demonstrated that eight transmission levels could be obtained by switching a GST cell on SiN waveguides [185]. The switching was attained by evanescent coupling between the GST cell and the light travelling along the waveguide as a result of optical pumping of the GST cell. This work also showed wavelength-selective operation at three different wavelengths, using microring resonators of various radii. Recently, this multilevel photonic memory has been improved and is now capable of storing up to 34 nonvolatile reliable and repeatable levels (around 5 bits) [190], see Figure 8a. Analogous to the work developed by Ríos et al. [185], exploiting the evanescent part of the optical mode which goes along a SiN waveguide and interacts with the deposited GeTe PCM on top of the waveguide [191], reversible phase modifications between amorphous and crystalline states were demonstrated in [192], showing changes in both the optical transmission and the resistance of the nanowire, see Figure 8b. Moreover, a fully addressable memory cell in both electrical and optical domains was first shown in [184] as a result of a successful combination of GST with a partially etched SiN rib waveguide-integrated plasmonic nanogap, Figure 8c. The plasmonic nanogap was formed between two metal electrodes, where a thin film of GST bridged the nanogap to control optical transmission and electrical resistance of the device, depending on the different states of GST.

GST was also integrated onto a SiN photonic crystal nanobeam cavity, enhancing the contrast and decreasing the energy consumption compared to the conventional waveguide design due to the larger group index of the cavity [193], see Figure 8d. In [194], the thermo-optic coefficients of the crystalline and amorphous state of GST were extracted, both the real and imaginary parts. In [195], the central element of an all-optical calculator, a photonic abacus was demonstrated, Figure 8e. Later on, a tensor core capable of operating at speeds of order 10${}^{12}$ of multiply–accumulate operations per second with a bandwidth exceeding 14 GHz was demonstrated in [196], exploiting SiN waveguides with GST as on-chip matrix multiplication. In [197], a broadband photonic tensor core with integrated ultralow crosstalk wavelength multiplexers was shown, as seen in Figure 8f. This approach proves simultaneous computing and data transfer at speeds comparable to fibre networks, overcoming the drawbacks from the analogous approaches which were lacking compared to the nonvolatile PCM-based memories [189,198,199].

The advantages and disadvantages of integrating GST on both the Si and SiN platforms in the C- (1530–1565 nm) and L- (1565–1625 nm) bands were precisely studied in [200]. While different PCM-based silicon and SiN building blocks have generally been evaluated in the C-band [188,201], Faneca et al. first evaluated the optical performance of an N-rich silicon nitride Mach–Zehnder interferometer (MZI) based on GST in the O-band for optical communications and reported a satisfactory extinction ratio of 11 dB between the amorphous and the crystalline states of the MZI [202]. Later, they also compared and investigated the optical properties of GST-based silicon nitride straight and RR waveguides in the O- and C-bands [187]. In the case of the straight waveguides, when the GST cell was switched between the two states, the researchers observed a high transmission contrast of 2.5 dB/μm in the C-band and 6.4 dB/μm in the O-band. Furthermore, high quality factor resonances (Q = $10^{5}$) were reported in both bands as a result of the GST deposition onto the RR waveguides.

Although GST is the material commonly used for nonvolatile PCM absorption modulation (amorphous phase GST 225: 0.039 dB/μm and crystalline phase: 2.7 dB/μm at 1550 nm [187]), it should be stated that GST is just one of the PCM materials available for nonvolatile photonic applications [191]. In fact, new PCMs with interesting optical properties are also emerging to complement GST [203]. As a result of the addition of selenium to GST, the component Ge_2_Sb_2_Se_4_Te_1_ (GSST) presents a moderately low-loss alternative to GST for different PCM-based building blocks. However, both GST and GSST still show a relatively high loss (extinction coefficient) when switched optically [203,204]. In [204], a reconfigurable and nonvolatile Bragg grating based on the combination of GSST with a SiN platform was proposed in the C-band and a Bragg resonance shift up to 15 nm, accompanied with a large amplitude modulation (insertion loss of 22 dB) was reported, see Figure 8g.

By taking advantage of a novel family of low-loss PCMs, which includes Sb_2_S_3_ and Sb_2_Se_3_ [205], a SiN Bragg grating design was also explored with Sb_2_S_3_, demonstrating low losses in both states and producing a 7 nm red-shift in the Bragg wavelength [204], see Figure 8g. Both Sb_2_S_3_ and Sb_2_Se_3_ exhibit lower inherent losses compared to GST and GSST, since their extinction coefficient is less than $10^{-4}$ in both states at 1550 nm and at 1310 nm [10,206]. The low optical loss and the optical phase modulation of Sb_2_S_3_ were investigated by Dong et al. onto the SiN platform at 750 nm [203], showing the potential of the PCM broadband material together with a silicon nitride platform in applications such as, postfabrication trimming, large-scale integrated quantum photonic networks and optical field-programmable gate arrays (FPGAs) in the visible range of the spectrum. In [207], low-loss PCMs (Sb_2_S_3_ and Sb_2_Se_3_) were integrated with MZI building blocks based on a SiN platform, see Figure 8h, to experimentally demonstrate the advantages of using this platform integrated with PCMs in both the O- and C-bands, in comparison with a SOI platform [208]. This experiment demonstrated a low insertion loss of around 0.04 dB/μm for Sb_2_S_3_- and 0.09 dB/μm for Sb_2_Se_3_-integrated devices in both amorphous and crystalline states. The change shown in the effective refractive index for Sb_2_Se_3_ was 0.03 at 1310 nm and 0.05 at 1550 nm, whereas for Sb_2_S_3_, it was 0.05 at 1310 nm and 0.02 at 1550 nm.

For switching higher volumes of PCM to achieve a higher amplitude or phase modulation, electrical switching with external heaters is required due to the high switching power needed in the optical domain for recrystallisation [186,206] and due to crystallisation filamentation and nonhomogeneous heating in the classical electrical switching [209]. Because silicon can be easily doped, the design of silicon-based heaters for nonvolatile photonic integrated circuits have been demonstrated in [210]. In a silicon nitride platform, doping the dielectric layer to use it as a heater is nontrivial and requires a high amount of implantation to obtain a tiny effect on the layer [211,212], making this option a difficult solution. To overcome this drawback, Fang et al. proposed the implementation of a layer of indium tin oxide (ITO) as an external transparent heater to switch the PCM phase between different states on SOI-based ring resonators [203], achieving an extinction ratio of over 30 dB near 1550 nm, proving the compatibility of ITO as a heater for future PCM-based SiN building blocks. Lately, graphene heaters are emerging as a possible solution to control the PCM temperature in photonic integrated circuits [213,214,215], see Figure 8i. Even though a relatively large amount of progress has been achieved in ITO and doped silicon-based heaters, graphene can reduce the switching energy and increase the speed maintaining a relatively low loss and the compatibility with the SiN platform [214].

### 5.2. Permanent Tuning of the Refractive Index

The refractive index of silicon nitride deposited by PECVD can be varied from 1.7 up to 3.1, depending on the ratio between its silicon and nitrogen content [10,216]. This value is determined during the deposition process, but in the case of nitrogen-rich silicon nitride (N-rich SiN) (with refractive index of 1.9), it can also be permanently varied postfabrication, offering a higher level of tunability.

It has been proven that the refractive index of N-rich SiN decreases when exposed to ultraviolet light [217]. Using a laser as the light source, the magnitude of the refractive index change can be controlled by varying the exposure time and the laser power. Thanks to the high power density and directionality of a laser beam, this process can be much quicker compared to using an incoherent source, and can be localised to a specific area.

By exposing a section of an RR for a few seconds (or some minutes, depending on its free spectral range and on the power of the laser), it is possible to switch its output, shifting its resonances towards shorter wavelengths (blue shift). Experimentally, a shift of −6.95 nm of the resonant wavelength has been demonstrated after exposing with a UV laser ( 244 nm wavelength and 40 kJ/cm${}^{2}$ fluence) a 126 μm long section of an RR with FSR of 6.15 nm [218].

This method can be applied to correct fabrication variations and for the reconfiguration of photonic circuits based on RRs, or other coupling devices such as splitters based on Mach–Zender interferometers. This technique has the advantage of providing a low-cost fine-tuning postprocessing method since no additional fabrication steps are required. Furthermore, a real-time adjustment of any photonic devices on the chip is possible during the exposure, enabling a high accuracy of trimming.

Similar results can be obtained by depositing a photosensitive polymer layer on top of the waveguides [219,220]. Localised thermal annealing of the silicon dioxide cladding enables a permanent trimming of the response of SiN ring resonators as well [221]. These methods are compatible with any type of SiN, but because of their additional fabrication complexity, the advantages of the laser-trimming technique are lost.

## 6. III-V/SiN Integration: Towards Efficient Monolithic Lasers on Silicon Substrate

**Figure 9:**
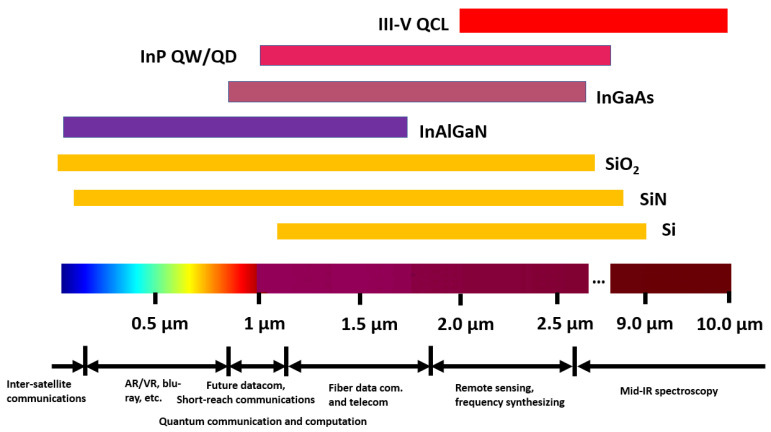
Spectral transparency of SiO_2_, Si and SiN (yellow); bandgap of III-V materials and gain structures (purple to red) and representative applications.

The explosive growth of silicon-based photonic integrated circuits (PICs) along with their wide applications requires high-performance coherent light sources lasers. Current commercialised silicon PICs are mainly powered by external free-running semiconductor lasers, [222,223] substantially increasing the cost of the systems, the power requirements and limiting scalability. In order to satisfy a range of systems, the integration of lasers to CMOS PICs requires a number of specifications such as a high output power, low lasing threshold, high temperature stability, ultralow linewidth and low noise level. Because of the indirect bandgap of Si, the heterogeneous or monolithic integration of lasers based on direct-bandgap III-V materials is currently the preferred solution for silicon PICs [224]. This is despite substantial progress related to the lasers based on group-IV SiGeSn materials which have been demonstrated at low temperatures and mid-infrared wavelengths [225,226,227]. Currently, most III-V laser integration schemes are based on the silicon-on-insulator (SOI) platforms [228,229], focusing on wavelengths in the near- and mid-infrared (NIR and MIR) regions, owing to the transparency window of Si (Figure 9). In contrast, silicon nitride (SiN) possesses a wider transparency window, extending from the MIR down to the UV, due to the large bandgap of the material. This matches the available bandgap of III-V semiconductor families (Figure 9). As discussed previously, due to the SiN benefits from an extremely low thermo-optic coefficient [230,231], nonlinear coefficient [232], tunable refractive index (by adjusting the Si/N ratio) [10,216] and CMOS-fabrication compatibility, ultralow-loss (<0.1 dB/cm) SiN is quickly becoming an ideal candidate for a range of applications in silicon photonics [8,41]. In contrast, III-V lasers integrated with SiN waveguides (III-V/SiN lasers) have yet to be developed fully, to compete with the widely reported III-V/Si lasers [228,229]. In this section, we focus on the recent progress achieved in III-V lasers (represented by GaN, InP and GaAs) along with their major characteristics required for CMOS PICs.

**Figure 10:**
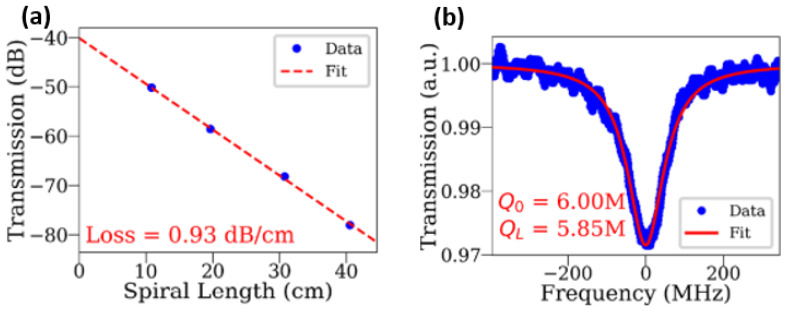
(**a**) Cutback measurement results for 0.8 μm wide waveguide spirals of around 405 nm and (**b**) measured transmission spectra of a high-Q mode at 453 nm; reprinted with permission from [240], ©2021 The Optical Society.

As the most obvious material advantage compared with that of Si-, SiN-coupled lasers can extend working wavelengths down to the visible–NIR wavelength range below 1100 nm, where virtual reality (VR)/augmented reality (AR), high-density optical storage, short-reach communication, and quantum technologies play important roles (Figure 9) [233,234,235]. An example of the benefits offered by integration in SiN was demonstrated by Kumari and colleagues, who employed a SiN-based high-index-contrast subwavelength grating (HCG) to substitute one of the distributed Bragg reflectors (DBRs) of a GaAs VCSEL working at 850 nm, via adhesive die bonding [236]. The grating structure not only worked as a cavity mirror, but it also guided light into the horizontal direction (perpendicular to the DBR stacks) out of the cavity. Following this, they fabricated an intracavity polarisation-insensitive HCG coupler followed by a SiN waveguide. By adjusting the parameters of the grating (duty cycle/period) and the oxide aperture size (4–6 μm), the laser exhibited a low threshold of only 1.1 mA and maximum single mode suppression ratio (SMSR) of 55 dB at ~845 nm [237,238,239]. At a shorter wavelength range, a violet-blue (405–435nm) RR with high quality factor (Q) up to $6\times 10^{6}$ using low-loss ( 0.9 dB/cm) SiN waveguides have been fabricated (Figure 10a,b) [240], and a conceptual blue III-Nitride/SiN PIC has been proposed by employing vertical grating couplers (with a maximum simulated coupling efficiency of 40%) and adiabatic optical tapers (with simulated coupling loss <4 dB), respectively, [241]. In addition to the progress in the visible–NIR wavelength range, SiN also demonstrates its versatility in the MIR region, where photonic devices and integrated systems with performances comparable to their Si counterparts have been developed [242]. Nevertheless, III-V/SiN lasers targeting the MIR region have not been reported so far, which is in contrast with progresses reported on the development of MIR III-V/Si lasers [242,243]. Besides a wider transparency window, III-V/SiN-coupled lasers also have a better thermal stability compared with III-V/Si lasers, which is crucial for the wavelength tuning stability in PICs. For example, Iadanza and colleagues demonstrated a single mode laser (with SMSR = 45 dB) with a III-V gain chip coupled to an external SiN DBR waveguide [244]. The laser exhibited extreme thermal stability with mode-hop-free behaviour under an injection current range of 15–62mA from room temperature up to 80 °C. The authors attributed the improved lasing properties to the low thermal-optical coefficient of SiN [230,231]. The above-mentioned reports on III-V/SiN lasers with excellent photonic characteristics prove that they can bring added functionalities of CMOS PICs to III/Si lasers, by extending the working wavelength to a wider range.

**Figure 11:**
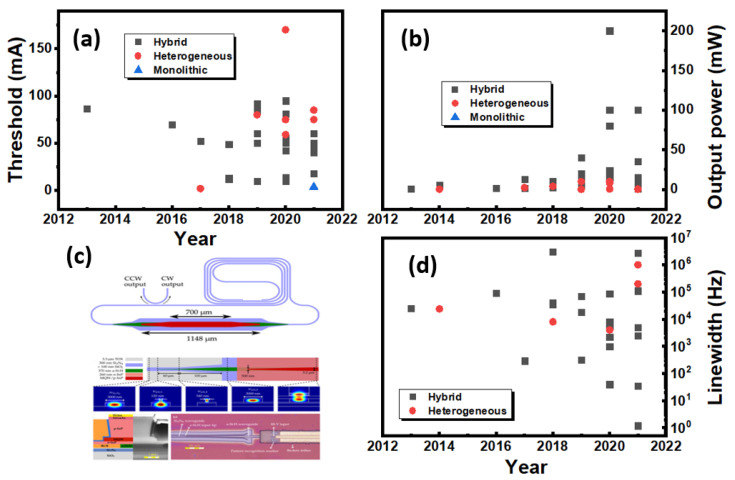
Development of (**a**) threshold current and (**b**) maximum output power of III-V/SiN-coupled lasers [239,244,245,246,247,248,249,250,251,252,253,254,255,256,257,258,259,260,261,262,263,264,265,266,267,268,269,270,271,272,273,274,275,276,277,278,279,280], (**c**) schematic layout of a heterogeneous III-V/SiN laser and its optical taper between SiN and III-V gain sections [245] and (**d**) development of linewidth of III-V/SiN lasers [244,246,248,249,252,254,256,257,258,261,262,263,264,266,267,270,272,273,274,275,276,279,280,283,284,285].

Figure 11a shows the recent progress related to the integration methods of on-chip lasers coupled with SiN waveguides [239,244,245,246,247,248,249,250,251,252,253,254,255,256,257,258,259,260,261,262,263,264,265,266,267,268,269,270,271,272,273,274,275,276,277,278,279,280]. Except for those labelled in Figure 11a,b, almost all lasers are coupled to the SiN platform with mature or commercial InP gain structures (DFB, reflective semiconductor optical amplifier, FP, etc.) [281] by hybrid or heterogeneous integration. In general, the hybrid integration, utilising easily controlled edge-/butt-coupled schemes to merge bulky SiN platforms and a separate III-V gain chip, gives a better threshold and output power (Figure 11a,b), while the III-V gain is often homoepitaxially grown on a non-Si substrate. This method often leads to a large footprint not favoured by CMOS PICs. Both heterogeneous (wafer-/die-level gain area transfer) and monolithic (III-V grown directly on Si) integration are more promising, even though they are at an early stage of maturity. It is worth to mention that sophisticated optical couplers are often required for heterogeneous/monolithic III-V lasers owing to the large difference in the refractive index between SiN and III-Vs (>1.1 at 1550 nm). Figure 11c shows an optical taper that consists of a III-V/Si intermediate layer/SiN layer used in a single-frequency laser, to couple light from the gain region into SiN ring waveguides, generating a threshold current of 59 mA and on-chip output power of 0.35 mW [245]. Such couplers, developed since the first heterogeneous III-V/SiN laser [265], are subject to coupling losses from sub-dB to several dB and can be further improved by structural design and material engineering. It is also noticeable that a majority of integrated lasers in recent years are based on InP (Figure 11a,b) [281], regardless of the fact that GaAs lasers have a higher quantum efficiency and a better thermal conductivity. Recent advances in telecom-wavelength lasing based on high-gain GaAs QDs heteroepitaxially grown on Si and SOI substrates with low thresholds and high temperature stability [282], indicate that it is a promising gain material for III-V/SiN monolithic integration and there is a strong possibility that 100 mW level heterogeneous/monolithic III-V/SiN lasers are achievable in the near future.

The narrow linewidth of a laser that is often associated with low noise, is an important metric for the integration with the Si PICs towards practical applications in communications [286], LIDAR [287], spectroscopy [288] and optical clocks [289]. Traditional DBR or DFB lasers utilise intracavity feedback from the cavity mirrors, which normally realise a megahertz-level linewidth [290,291]. In contrast, a III-V/SiN-coupled laser is a natural external cavity configuration, whose lasing dynamics can be analysed by the same theory as that of an external cavity semiconductor laser [292], where the linewidth reduction is one of the major advantages. The linewidth of an external cavity laser can be calculated from, $\Delta \omega =\frac{\Delta \omega_{0}}{1+A+B}$, where $\Delta \omega_{0}$ is the cavity linewidth, *A* and *B* are parameters related to phase (increasing group delay and photon lifetime) and spectral feedback of the external cavity (related threshold gain and local carrier density) [293]. Benefiting from the fast-developing SiN devices, especially resonators with Q up to >200 M [294], a highly precise phase (factor A) and spectral (factor B) feedback [295,296] is achievable in addition to an extended cavity length (thereby reducing $\Delta \omega_{0}$), likely to generate ultranarrow linewidth. Figure 11d displays the state-of-the-art linewidth of III-V/SiN-coupled lasers in terms of integration method [244,246,248,249,252,254,256,257,258,261,262,263,264,266,267,270,272,273,274,275,276,279,280,283,284,285]. It appears that significant progress has been made with hybrid integration when compared to heterogenous/monolithic integration, though the latter started its development at a later stage. The best results reported so far show that a hertz-level linewidth on a III-V/SiN laser is achievable with hybrid integration [283], which is the record value regarding III-V and Si photonics integrated platforms. The authors utilised ultrahigh Q (170 M–270 M) microresonators externally coupled to a III-V DFB laser based on high-aspect-ratio SiN waveguides ( 100 nm in thickness and 2.8 μm–8 μm in width). By precisely controlling the feedback phase from the SiN resonator to the active region (to adjust the air gap between chips), self-injection-locking was achieved. They observed a record narrow linewidth of 1.2 Hz along with low frequency noise of 0.2 Hz${}^{2}$/Hz. Though this is an example of a hybrid integrated laser, ultralow linewidth with low noise could also be achievable using heterogenous/monolithic III-V/SiN-coupled lasers with a smaller footprint, given that the technical barriers only lie in the III-V crystal quality on Si and the III-V/SiN interface engineering.

In summary, we reviewed the progress of III-V/SiN lasers associated with their performance and fabrication methods. The III-V/SiN-coupled lasers have arisen as a strong competitor to III-V/Si lasers, due to their advantages in terms of their extended transparency window down to UV–visible range and their better thermal stability. Based on the above discussions, it is concluded that the state-of-the-art integration technologies of III-V/SiN lasers are at very different stages. To date, the III-V/SiN laser with high performance (e.g., low threshold, high output power, narrow linewidth) are mainly fabricated by hybrid or heterogeneous integration with a relatively large footprint and some back-end type CMOS compatibility. The more recent and still challenging monolithic integration is also gaining attention, [259,260,297] and has a great potential for dramatically reducing the costs by fully leveraging the CMOS economy of scale and pushing further the limits of very large scale integrated photonics (VSLI-P) [298].

## 7. Conclusions

Silicon nitride has been a key material in the CMOS industry as well as a material for stand-alone optical circuitry for decades. More recently with the advent of silicon photonics and CMOS PICs, silicon nitride has generated a substantial interest in many areas of research and applications to complement the more “standard” silicon platform. As discussed in this review, silicon nitride displays a plethora of capability such as waveguide loss in the order of 1 dB/m, stability at high optical power level, low thermo-optic coefficient, highly efficient nonlinear operations, integrability with material-enabling modulation and lasing schemes on par or better than when using silicon-based devices, and it offers transparency from UV to MIR. Furthermore, silicon nitride is a flexible material through strain and refractive index engineering and can be used as a back-end process for photonic integration purposes, as a transition layer potentially providing a pathway for monolithic integration of a range of materials and devices on a CMOS based PIC. It is therefore clear that silicon nitride is an enabling material key to CMOS electronics and photonics and through the demonstration of these photonic building blocks it will enhance further the underpinning technology that is CMOS photonics. 

## Figures and Tables

**Table 1:** Low-loss LPCVD SiN fabrication processes.

Fabrication	Deposition	Material	Core	C-Band	Substrate
Approach	Method		(nm${}^{2}$)	( dB/cm )	(mm)
Thermal cycling [22,24,25]	LPCVD	Si_3_N_4_	1800 × 910	0.04 (1550 nm)	100
Photonic Damascene reflow [21,27,28,29]	LPCVD	Si_3_N_4_	2000 × 600	0.05 (1550 nm)	100
Multistep	LPCVD	Si_3_N_4_	1800 × 645	0.03 (1550 nm)	76
Annealing [31,32]		Si-rich SiN	1850 × 600	0.40 (1550 nm)	
Twist-and-grow [33,34,35]	LPCVD	Si_3_N_4_	1600 × 800	0.04 (1560 nm)	200

**Table 2:** Comparison of different Si-rich SiN platforms.

Material [Reference]	Refractive Index (@1550 nm)	Deposition Method	Core (nm${}^{2}$)	Propagation Losses (@1550 nm) (dB/cm)	Kerr Coefficient $n_{2}$ (m^2^/W)
Si-rich SiN [32]	2.07	LPCVD (annealing)	1800 × 645	0.4	0.6 × 10${}^{-18}$
Si-rich SiN [39]	2.49	PECVD	1000 × 297	1.5	1.61 × 10${}^{-18}$
Si-rich SiN [39]	2.71	PECVD	1000 × 308	6	2 × 10${}^{-18}$
USRN [43]	3.1	PECVD	550 × 300	4.5	28 × 10${}^{-18}$
USRN [48]			450 × 330	3	

**Table 3:** Characteristics of the deposited Si-rich SiN layers used for the nonlinear performance study. Reproduced from [39] under a CC BY 4.0 license.

Layer ID	Refractive Index (@1550 nm)	Thickness (nm)
01	2.01	300
02	2.49	297
03	2.71	308

**Table 4:** Imγ coefficients measured by means of pulse-transmission experiments for different Si-rich SiN layer compositions and different waveguide widths. Reprinted, with permission, from [41], ©2019 IEEE.

Layer ID	Imγ (Wm${}^{-1}$) W = 500 nm	Imγ (Wm${}^{-1}$) W = 700 nm	Imγ (Wm${}^{-1}$) W = 1000 nm
01	Negligible	Negligible	Negligible
02	Negligible	Negligible	Negligible
03	1.75	1.44	1.11

**Table 5:** The characteristic comparison of fabricated EO-polymer modulators based on SiN waveguide.

Modulator Type	Modulation Frequency	Modulation Figure of Merit (Vπ · L or Vπ)
Phase modulator (2016) [82]	1 kHz	900 V·cm
Phase modulator (2015) [95]	-	17.6 V
Ring resonator (2008) [96]	10 GHz	-

**Table 6:** State-of-the-art performance matrix for the fabricated high-speed modulators in the SiN platform. The table includes reported results for C- communication band.

Configuration	Platform	Modulation Bandwidth (GHz)	Modulation Efficiency	Loss (dB/cm)
Ring resonator [82]	SiON or SiN & EO polymer	10	-	0.8
Mach–Zehnder interferometer [106]	PECVD Si-rich SiN & LN	100	3.1 V·cm	0.2
Ring resonator [84]	PECVD SiN & BTO	-	0.3 V·cm	9.4±0.4
Ring resonator [132]	PECVD SiN & Graphene	30	1.5 dB/V	-
Ring resonator [144]	LPCVD SiN & MoS${}_{2}$	-	0.09 V·cm	-
